# Rhes influences striatal cAMP/PKA-dependent signaling and synaptic plasticity in a gender-sensitive fashion

**DOI:** 10.1038/srep10933

**Published:** 2015-07-20

**Authors:** Veronica Ghiglieri, Francesco Napolitano, Barbara Pelosi, Chiara Schepisi, Sara Migliarini, Anna Di Maio, Valentina Pendolino, Maria Mancini, Giuseppe Sciamanna, Daniela Vitucci, Giacomo Maddaloni, Carmela Giampà, Francesco Errico, Robert Nisticò, Massimo Pasqualetti, Barbara Picconi, Alessandro Usiello

**Affiliations:** 1 Department of Philosophy, Human, Social, and Educational Sciences, University of Perugia, Perugia, Italy; 2 Fondazione Santa Lucia IRCCS, Rome, Italy; 3 CEINGE Biotecnologie Avanzate, Naples, Italy; 4 Department of Molecular Medicine and Medical Biotechnology, University of Naples “Federico II”, Naples, Italy; 5 Department of Biology, University of Pisa, Pisa, Italy; 6 Department of Physiology and Pharmacology, University of Rome “La Sapienza”, Rome, Italy; 7 Department of Systems Medicine, University of Rome “Tor Vergata”, Rome, Italy; 8 Dipartimento di Scienze Motorie e del Benessere DiSMeB, University of Naples “Parthenope”, Naples, Italy; 9 Istituto Italiano di Tecnologia, Center for Neuroscience and Cognitive Systems, Rovereto, Italy; 10 Department of Environmental Sciences, Second University of Naples, Naples, Italy

## Abstract

Mechanisms of gender-specific synaptic plasticity in the striatum, a brain region that controls motor, cognitive and psychiatric functions, remain unclear. Here we report that *Rhes,* a GTPase enriched in medium spiny neurons (MSNs) of striatum, alters the striatal cAMP/PKA signaling cascade in a gender-specific manner. While *Rhes* knockout (KO) male mice, compared to wild-type (WT) mice, had a significant basal increase of cAMP/PKA signaling pathway, the Rhes KO females exhibited a much stronger response of this pathway, selectively under the conditions of dopamine/adenosine-related drug challenge. Corticostriatal LTP defects are exclusively found in A2AR/D2R-expressing MSNs of KO females, compared to KO males, an effect that is abolished by PKA inhibitors but not by the removal of circulating estrogens. This suggests that the synaptic alterations found in KO females could be triggered by an aberrant A2AR/cAMP/PKA activity, but not due to estrogen-mediated effect. Consistent with increased cAMP signaling, D1R-mediated motor stimulation, haloperidol-induced catalepsy and caffeine-evoked hyper-activity are robustly enhanced in Rhes KO females compared to mutant males. Thus Rhes, a thyroid hormone-target gene, plays a relevant role in gender-specific synaptic and behavioral responses.

The Ras homolog enriched in striatum (Rhes) is a small GTP-binding protein discovered by subtractive hybridization^1^ and highly expressed throughout the dorsal striatum and nucleus accumbens of rodent brain^2^,^3^,^4^. *Rhes* transcription is regulated by thyroid hormones during development^4^,^5^,^6^,^7^ and by dopamine (DA) in the adult rat brain^8^.

Early studies in cell lines^3^,^9^ indicated that Rhes, most likely by binding to G_α_i, reduces G-protein-coupled receptor (GPCR)-mediated accumulation of cAMP^10^. Accordingly, *Rhes* knockout (KO) mice have increased striatal cAMP/PKA activity^2^. In addition to its influence on cAMP accumulation, Rhes acts as a selective striatal E3-ligase of mutant huntingtin, the sumoylation of which promotes neurotoxicity^11^,^12^,^13^. These *in vitro* observations together with *in vivo* findings^14^ are consistent with the hypothesis that Rhes-mediated functions are common striatal targets for selective neurodegeneration in basal ganglia disorders^15^.

Consistently, it has been found that Rhes mediates AKT-mediated signaling^16^,^17^, participates in intracellular iron homeostasis^18^ and, most notably, modulates mammalian target of rapamycin complex 1 (mTORC1). This critical pathway is associated, among other processes^19^, with L-DOPA-induced dyskinesia (LID) in animal models of Parkinson disease^20^,^21^. These findings support the hypothesis that putative anti-Rhes drugs may attenuate the neurological manifestations of Huntington disease and LID^22^,^23^. Despite its growing role in striatal pathologies, the function of Rhes GTPase in striatal physiology remains less understood.

Previous studies indicated that lack of Rhes resulted in sex–sensitive behavioral phenotypes in mutants^24^,^25^, but the mechanisms are unknown. Here we dissected the mechanisms of the gender-specific differences associated to *Rhes* deletion, focusing on striatal cAMP/PKA-dependent signaling and tested the differential motor responses elicited by DA and adenosine agonist/antagonists. Overall, our study indicates that Rhes orchestrates gender-sensitive alterations in striatal signaling, synaptic plasticity, and behavioral responses.

## Results

### *Rhes mRNA* expression in GABAergic medium spiny neurons of mouse and human striatum

**Figure 1:**
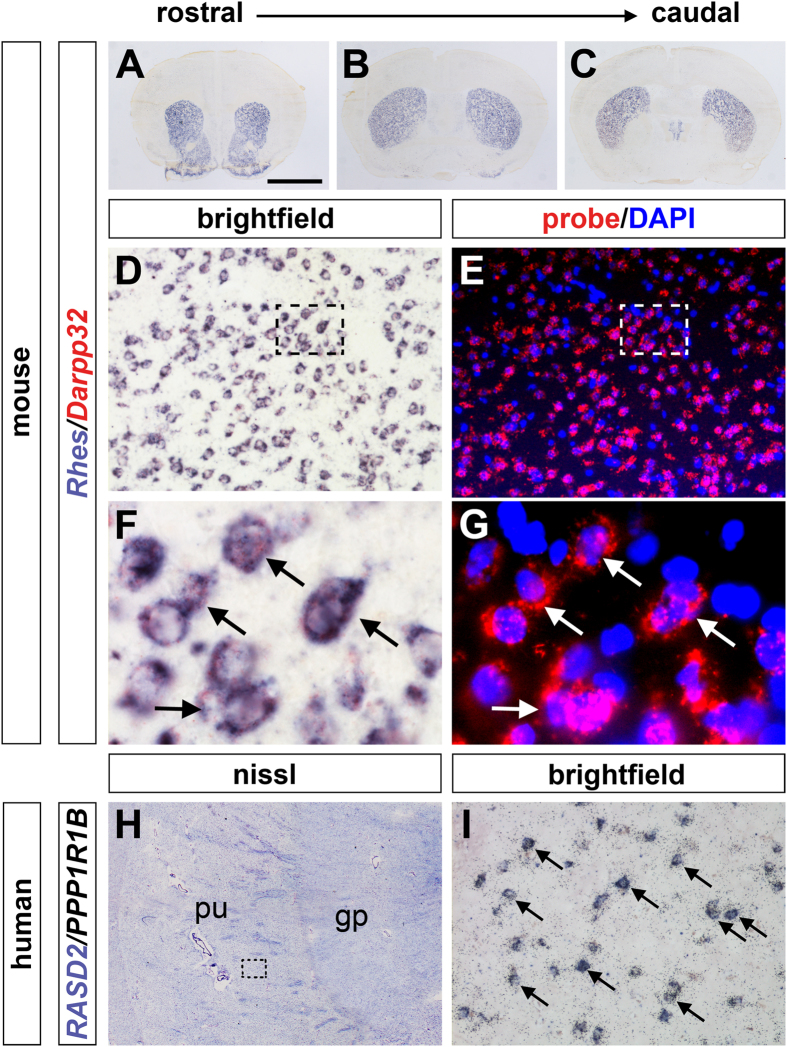
*Rhes mRNA* expression in both mouse and human striatal MSNs. (**A**–**C**) *In situ* hybridization (ISH) of coronal sections showing *Rhes* expression along the rostro-caudal extent of the mouse striatum. (**D**–**G**) Double ISH of mouse caudate/putamen (CPu) using specific antisense riboprobes for *Rhes* (**D,F**) and *Darpp32* (**E,G**), showing that *Rhes* is expressed in *Darpp32*-positive striatal MSNs. Boxed regions in (**D,E**) are shown at higher magnification in both brightfield (**F**) and fluorescence (**G**), and neurons strongly expressing both *Rhes* and *Darpp32* are indicated by arrows. (**H,I**), Double ISH analysis of *RASD2* and *PPP1R1B* expression on cryosections from post-mortem human brain. (**H**) Nissl stained specimen highlighting the neuroanatomical region of the putamen and globus pallidus nuclei. (**I**) Brightfield photomicrograph image of the boxed region in (**H**) showing co-localizing *RASD2* (purple) and *PPP1R1B* (silver grains) mRNA expression in MSNs (arrows). Abbreviations: gp, globus pallidus nucleus; pu, putamen nucleus. Scale bar: 2.5 mm (**A–C,H**); 75 μm (**D,E,I**); 15 μm (**F,G**).

To identify the striatal neurons that express *Rhes,* we performed double *in situ*-hybridization (ISH) on coronal sections along the rostro-caudal extent of the striatum of wild-type (WT) mouse brains (Fig. 1A–C). As a marker of GABAergic medium spiny neurons (MSNs) we used protein phosphatase 1 regulatory subunit 1B (PPP1R1B), also known as Darpp32, a bifunctional signal transduction molecule, whose phosphorylation and function is regulated by dopaminergic and adenosine receptor stimulation^26^. As expected, *Rhes* expression was observed in virtually all *Darpp32*-positive striatal neurons, corresponding to the projecting MSNs (Fig. 1D–G, arrows). Then, we performed double ISH using *RASD2* and *PPP1R1B* riboprobes, respectively *Rhes* and *Darpp32* human orthologs, to analyze mRNA distribution of this GTPase in the MSNs of human striatum (Fig. 1H,I). Consistent with the results in mouse brain, we found that *RASD2* mRNA was highly enriched in the putamen nucleus and expressed in virtually all the *PPP1R1B*-positive projecting MSNs (Fig. 1I, arrows).

### Lack of Rhes unmasks a gender-dependent modulation of striatal cAMP/PKA signaling

**Figure 2:**
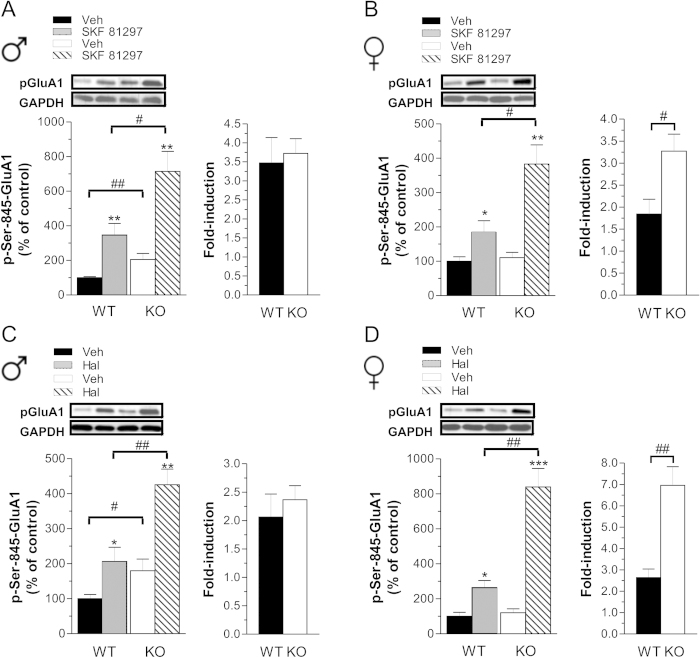
Effect of dopaminergic drugs on striatal cAMP/PKA activity in *Rhes* male and female mutant mice. (**A,B**) Striatal p-Ser845-GluA1protein levels, in male (**A**) and female (**B**) WT and KO mice, following 5 mg/kg SKF 81297 (WT, n = 9 males, n = 7 females; KO, n = 9 per gender) or vehicle (Veh) treatment (WT, n = 9/gender; KO, n = 9 males, n = 8 females) with relative fold-induction. (**C,D**) Striatal p-Ser845-GluA1 levels, of male (**C**) and female (**D**) WT and KO mice after 0.5 mg/kg haloperidol (Hal) treatment (WT, n = 8 males, n = 9 females; KO, n = 9/gender) or Veh (WT, n = 8 males, n = 9 females; KO, n = 9/gender), with relative fold-induction. Top panels show representative blots comparing the different genotypes and treatments. *p < 0.05, **p < 0.01, ***p < 0.0001, compared with Veh-treated group within genotype. #*p* < 0.05, ##*p* < 0.01, compared with WT group. All data are expressed as mean ± SEM. Genotypes are as indicated.

In order to evaluate whether the lack of Rhes modulates cAMP/PKA signaling in a gender-specific manner. we analyzed the phosphorylation state of a selective PKA substrate, the glutamate AMPA receptor subunit at Ser845 residue (p-Ser845-GluA1)^27^, in both male and female mutants versus their matched controls, under basal and challenged conditions. Administration of SKF 81297, a DA D1R full agonist, increased p-Ser845-GluA1 levels in males of both genotypes (Fig. 2A, left panel; two-way ANOVA, treatment effect: F_(1,32)_ = 29.844, *p* < 0.0001). Although SKF 81297 induced an apparently higher striatal p-Ser845-GluA1 level in mutants (*p* = 0.0145, Fisher’s *post-hoc*), its relative increase compared to baseline (expressed as fold-induction) was similar to WT animals (Fig. 2A, right panel). This observation suggests that lack of Rhes in males mainly perturbs striatal tonic cAMP/PKA activity, as indicated by the significantly higher basal p-Ser845-GluA1 levels in KO striata (*p* = 0.0092, Fisher’s *post-hoc*). Consistently, haloperidol, by unmasking the A2AR/cAMP/PKA-dependent signaling^28^ increased GluA1 phosphorylation in both WT and KO males (Fig. 2C, left panel; two-way ANOVA, treatment effect: F_(1,30)_ = 24.466, *p* < 0.0001). As seen with D1R agonist, despite the higher A2AR/cAMP/PKA signaling in mutants compared to treated-controls (*p* = 0.0026, Fisher’s *post-hoc*), we failed to find any significant difference in fold-induction between genotypes, due to the higher basal p-Ser845-GluA1 levels in KO (Fig. 2C, right panel; *p* = 0.0483, Fisher’s *post-hoc*). In contrast to what observed in male mutants, lack of Rhes in females did not result in altered basal striatal p-Ser845-GluA1 content (Fig. 2B,D, left panel). However, in KO females both SKF 81297 and haloperidol administration induced higher p-Ser845-GluA1 levels, when compared to WT treated controls (two-way ANOVA, genotype × treatment interaction, SKF 81297: F_(1,29)_ = 7.027, *p* = 0.0129; haloperidol: F_(1,32)_ = 22.383, *p* < 0.0001). As a consequence, in KO females, the comparable basal activity along with an exaggerated phasic D1R- and A2AR-dependent cAMP/PKA signaling resulted in a greater fold-induction of this pathway (Fig. 2B,D, right panels; SKF 81297, *p* = 0.0175; haloperidol, *p* = 0.0004). Overall, our findings indicate that lack of Rhes in females, rather than influencing the tonic striatal cAMP/PKA activity as seen in male mutants, selectively increases the phasic activation of this pathway.

### Lack of Rhes does not affect intrinsic membrane properties and corticostriatal long-term depression

**Figure 3:**
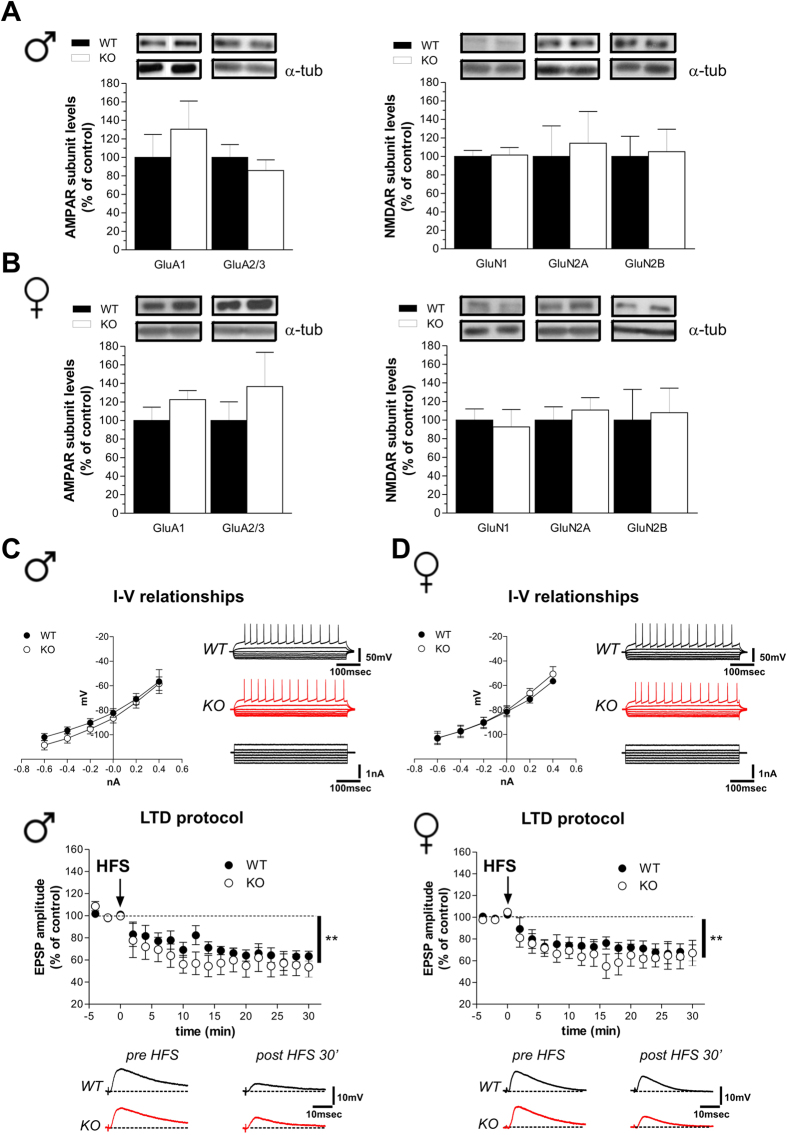
Intrinsic membrane properties and long-term depression in Rhes mutant mice. (**A,B**) Western blotting analysis on the striatal glutamate AMPAR and NMDAR subunit levels in male (**A**) (n = 6/genotype per GluA1, GluA2/3, GluN1, GluN2B; n = 5 WT, 6 KO per GluN2A), and female (**B**) (n = 6/genotype per GluA1, GluA2/3, GluN2A; n = 4 WT, 5 KO per GluN1; n = 6 WT, 5 KO per GluN2B) mice. The top panels show representative blots comparing the different genotypes, for each protein detected. All data are expressed as mean ± SEM. Genotypes are as indicated. (**C,D**) Current-voltage graphs (left) and representative traces (right) obtained after applying hyperpolarizing and depolarizing steps of current to MSNs recorded from WT (n = 6 males, n = 7 females) and KO (n = 10 per gender) mice. Time-courses (top) and example traces (bottom) of the post-synaptic responses measured in MSNs recorded from slices of male KO (n = 6) and WT (n = 5) mice (left panel) and female KO (n = 4) and WT (n = 5) mice (right panel), showing no difference in high-frequency stimulation (HFS)-induced LTD (Student’s *t*-test, pre- vs. 30 min post-HFS, **p < 0.01).

Based on the strict control of glutamatergic inputs on MSNs activity, we first analyzed the striatal AMPAR and NMDAR subunit expression in WT and KO animals. Western blotting analysis indicated that protein levels of both NMDAR (GluN1, GluN2A and GluN2B) and AMPAR (GluA1 and GluA2/3) subunits are not altered in knockouts of both genders (Fig. 3A,B; *p* > 0.05, per each protein). Next, we examined the effects of *Rhes* deletion in modulating synaptic properties and plasticity of striatal MSNs in mutant mice. Based on intracellular and patch-clamp recordings in corticostriatal slices, we failed to find any significant difference between genotypes in the intrinsic membrane properties of MSNs (Fig. 3C,D, upper panels; Student’s *t*-test, *p* > 0.05). Moreover, analysis of activity-dependent synaptic plasticity in striatal MSNs revealed that the delivery of a high frequency stimulation (HFS) to corticostriatal fibers in presence of Mg^2+^ (1.2 M) was able to induce comparable long-term depression (LTD) in the MSNs of both genotypes, without any evident effect of gender (Fig. 3C,D, bottom panels; two way ANOVA, gender effect, male: F_(1,136)_ = 0.71, *p > *0.05; female: F_(1,136)_ = 0.41, *p > *0.05).

### Lack of Rhes selectively affects corticostriatal long-term potentiation in A2AR/D2R-positive MSNs in a gender-specific manner

**Figure 4:**
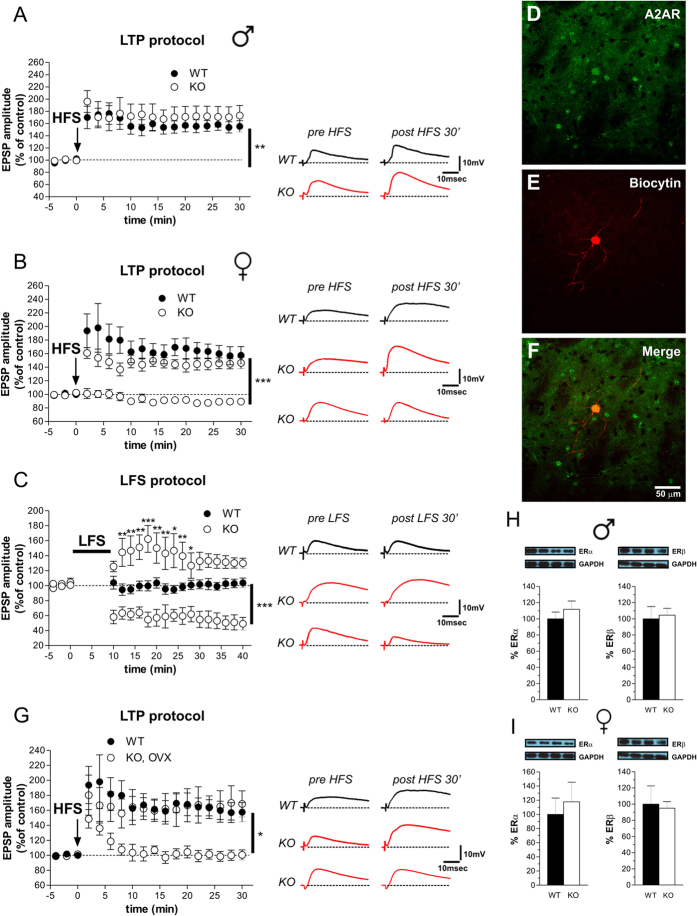
Long-term potentiation in MSNs of Rhes mutant mice and morphological characterization. (**A**) Time-course plots of EPSP amplitude (left) and pairs of traces (right) demonstrate the presence of HFS-induced LTP in MSNs recorded from slices of male WT (n = 5) and KO (n = 7) mice (Student’s *t*-test, pre- vs. 30 min post-HFS, **p < 0.01). (**B**) Time course graphs of the EPSC amplitude (left) and pairs of traces (right) show the induction (n = 12) or the complete loss (n = 15) of LTP in MSNs from female KO mice compared with age-matched WT animals (n = 5) (Student’s *t-*test at 30 min post-HFS, WT vs. KO with LTP, *p* > 0.05, WT vs. KO with no LTP, ***p < 0.001). (**C**) In KO females application of a low-frequency stimulation (LFS) induced a decrease of EPSP amplitude in 5 of 9 MSNs recorded and a mild potentiation in the remaining 4 MSNs, whereas the same protocol induced no change of synaptic activity in all the MSNs (n = 7) recorded from WT females (Bonferroni *post-hoc*: WT vs. KO with LTP, *p < 0.05; **p < 0.01; ***p < 0.001; Student’s *t-*test at 30 min post-HFS: WT vs. KO with a depression of EPSP, ***p < 0.001). (**D**–**F**) Confocal laser scanning microscopy (CLSM) images of double-labeled immunofluorescence for biocytin and adenosine receptor (A2AR). Biocytin immunolabeling is visualized in red streptavidin-Cy3 immunofluorescence (**D**) and A2AR is visualized in green Cy2 immunofluorescence (**E**). The colocalization of A2AR and biocytin is showed in merged panel (**F**) as yellow fluorescence. (**G**) HFS protocol applied in the MSNs of ovariectomized (OVX) KO females induced LTP in 6 of 12 MSNs recorded (Student’s *t-*test at 30 min post-HFS: WT vs. KO OVX with no LTP, *p < 0.05). (**H,I**) Striatal ERα and ERβ, determined by Western blotting, in WT and KO male (n = 6 /genotype per ERα; n = 6 WT, 5 KO per EPβ) (**H**) and female (n = 5 WT, 6 KO per ERα; n = 6 /genotype per ERβ) (**I**) mice. The top panels show representative blots comparing the different genotypes. All data are expressed as mean ± SEM. Genotypes are as indicated.

Then, we investigated the corticostriatal long-term potentiation (LTP) in male and female mutants. Delivery of HFS to corticostriatal fibers in Mg^2+^-free medium, a condition that favors NMDA receptor activation, was able to induce LTP with comparable amplitude and time course in WT and KO male mice (Fig. 4A; two-way ANOVA, genotype effect, *F*_(1,187)_ = 0.42, *p* > 0.05). Conversely, LTP amplitude was significantly altered in a subpopulation of MSNs recorded from mutant females. Specifically, although HFS-induced LTP was similar to controls in 12 out of 27 neurons, a complete loss of potentiation was found in the remaining 15 MSNs recorded (Fig. 4B; two-way ANOVA, genotype effect, F_(2,493)_ = 30.29, *p* < 0.001; time × genotype interaction, F_(34,493)_ = 8.71, *p* < 0.001).

To explain the synaptic plasticity defects found in KO females, we hypothesized that an exaggerated phasic cAMP/PKA activation associated to HFS might occlude LTP expression in MSNs that did not show any apparent activity-dependent plasticity. To test this idea, low frequency stimulation (LFS) protocol was applied to corticostriatal fibers during baseline in striatal slices of WT and KO females. While in WT this stimulation protocol did not induce any change in EPSP amplitude, as expected, in KO females LFS decreased the EPSP amplitude in 56% of the recorded MSNs. This abnormal synaptic depotentiation seen in female mutants was similar to that observed in control animals after the induction of a HFS-dependent LTP^29^. Further, in KO females, the remaining MSNs (44%) responded with a short-term potentiation. (Fig. 4C; two-way ANOVA, genotype effect, F_(2,234)_ = 19.51, *p* < 0.001; time × genotype interaction, F_(36,234)_ = 7.33, *p* < 0.001).

Finally, given the evidence of a heterogeneous population of striatal MSNs (SP/D1R-expressing direct and the A2AR/D2R-expressing indirect pathways)^30^, we performed post hoc immunohistochemical analysis to assess the identity of MSNs subtype in KO females not showing any apparent activity-dependent LTP. We found that the MSNs that did not respond to HFS with a physiological magnitude of LTP in KO females were adenosine A2AR-positive cells that belong to the D2R-expressing striato-pallidal pathway (Fig. 4D–F). Moreover, to determine whether circulating ovarian hormones could explain the gender-specific dichotomous response to HFS, we analyzed LTP in the MSNs of ovariectomized (OVX) KO females. Similar to what observed in intact mutants, HFS of corticostriatal fibers induced LTP only in 6 out of 12 MSNs recorded from OVX KO females (Fig. 4G; two-way ANOVA, time × genotype interaction, F_(17,170)_ = 4.90, *p* < 0.001). In addition, no significant difference was found in the striatal expression of Estrogen Receptor subunits (ERα and ERβ) between genotypes (Fig. 4H,I; *p* > 0.05 per each protein).

### Altered striatal LTP in Rhes mutant females in A2AR/D2R-positive neurons is associated to exaggerated cAMP/PKA signaling

**Figure 5:**
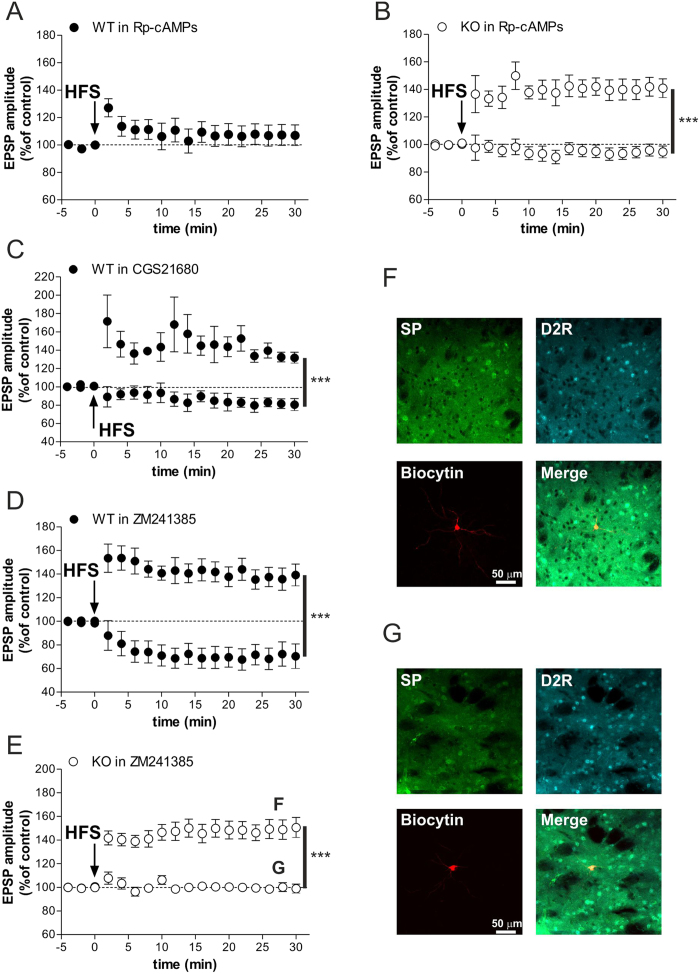
Altered striatal LTP in Rhes KO females associated to exaggerated A2AR/cAMP/PKA signalling. (**A**) The LTP of the MSNs recorded with intrapipette application of PKA inhibitor Rp-cAMPs was blocked in all the recorded cells (n = 4) (Student’s *t-*test at 30 min post-HFS: WT in Rp-cAMPs, pre vs post HFS, *p* > 0.05). (**B**) LTP induction in MSNs of KO females recorded with intrapipette application of Rp-cAMPs (Student’s *t-*test at 30 min post-HFS: KO with no LTP, n = 7, vs. KO with LTP, n = 5, ***p < 0.001). (**C**) bath application of A2AR agonist CGS21680 in slices from WT females revealed the existence of two different subsets of MSNs showing LTP and no LTP (Student’s *t-*test at 30 min post-HFS: WT with no LTP, n = 5, vs. WT with LTP, n = 4, ***p < 0.001). (**D**) A2AR antagonist ZM241385 bath applied in slices of WT females induces LTP in n = 12 cells and LTD in the remaining n = 6 neurons (Student’s *t-*test at 30 min post-HFS: WT with LTD vs. WT with LTP, ***p < 0.001). (**E**) ZM241385 application in KO neurons induced a regular LTP (n = 16) in SP/D1R-positive MSNs and no change (n = 10) in A2A/D2R-positive MSNs (Student’s *t-*test at 30 min post-HFS: KO with no LTP vs. KO with LTP, ***p < 0.001). (**F**,**G**) Confocal laser-scanning microscopy images of triple-labeled immunofluorescence for biocytin, SP and D2 receptor. SP is visualized in green, D2R is visualized in light blue-Cy5 fluorescence and biocytin immunolabeling is visualized in streptavidin-Cy3 fluorescence, the merged image is shown on the bottom right of the panels.

In light of our biochemical studies, we postulated that the excessive phasic cAMP/PKA signaling induced by HFS in female mutants might prevent the normal LTP expression in A2AR/D2R-positive MSNs. To verify this idea, we explored in WT and KO female striatal slices the consequences of changes in cAMP/PKA activity and A2AR activation on corticostriatal LTP expression. As expected, in WT slices patch-clamp recordings of MSNs showed that intrapipette application of PKA inhibitor Rp-cAMPs (100 μM) was able to block LTP^31^, in all the recorded cells (Fig. 5A), confirming that an intact cAMP/PKA activation is required for striatal HFS-induced LTP. In contrast, based on the hypothesis that an abnormal high A2AR/cAMP/PKA activity prevented HFS-induced LTP in Rhes KO females, we demonstrated that intrapipette application of a PKA inhibitor, at a dose that blocked LTP in all the MSNs of WT, was able to normalize synaptic plasticity in SP-negative MSNs (data not shown) of mutant females (Fig. 5B; two-way ANOVA, group effect, F_(1,170)_ = 40.53, *p* < 0.001). We next evaluated whether non-physiological A2AR activation in WT could affect the induction of LTP. To this aim, bath application of A2AR agonist (CGS21680, 3 μM) 10 minutes prior to HFS protocol revealed the existence of two different subsets of MSNs, one normally responding to HFS with a LTP amplitude similar to control, and the other being defective in this form of synaptic plasticity (Fig. 5C; two-way ANOVA, group effect, F_(2,119)_ = 12.83, *p* < 0.01). Therefore, the dichotomous MSNs behaviors under A2AR agonist strengthened the idea that, differently from SP/D1R-positive MSNs, non-physiological increase of cAMP/PKA signaling in A2AR/D2R-bearing neurons prevents its activity-dependent plasticity, as seen in Rhes KO females.

To further assess the role of A2AR activation on striatal LTP expression, we recorded the effects of the A2AR antagonist ZM241385 (1 μM) in WT slices. Intracellular and patch-clamp recordings showed that following blockade of A2AR, LTP was induced only in SP/D1R-positive MSNs (Fig. 5D). In contrast, the remaining A2AR/D2R-positive MSNs, which not responded with LTP, paradoxically showed a LTD (Fig. 5D; two-way ANOVA, group effect, F_(1,272)_ = 26.78, *p* < 0.001). Similar to what observed in WT slices, application of ZM241385 in KO females did not affect a regular HFS-induced LTP in SP/D1R-positive MSNs (Fig. 5F). In contrast, ZM241385 in A2AR/D2R-positive MSNs (Fig. 5G) of KO females failed to induce LTD (Fig. 5E; two-way ANOVA, group effect, F_(1,408)_ = 28.27, *p* < 0.001), unlike reported in controls.

### Rhes mutants display altered motor responses to dopamine and adenosine drugs in a gender-specific fashion

**Figure 6:**
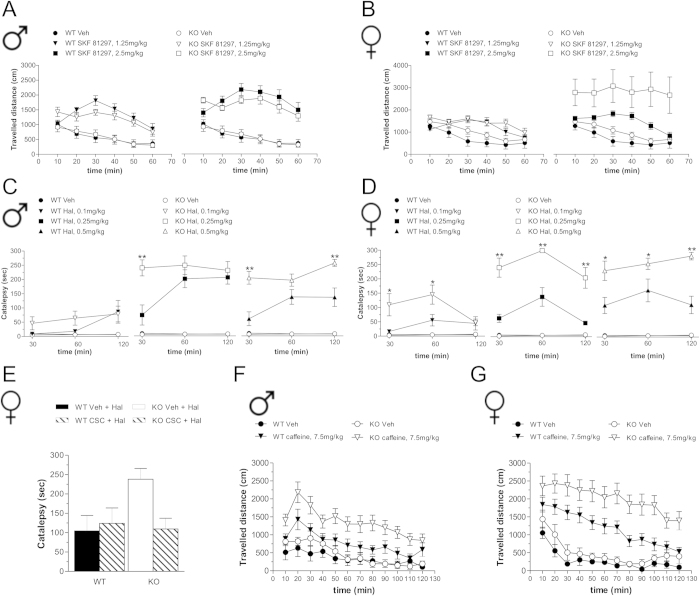
D1R and A2AR-dependent behavioral responses in *Rhes* mutant mice. (**A,B**) Locomotor activity induced in male (**A**) and female (**B**) WT and KO mice by SKF 81297 (1.25 mg/kg: WT, n = 15 males, n = 14 females; KO, n = 13 males, n = 14 females; 2.5 mg/kg: WT, n = 21 males, n = 21 females; KO, n = 23 males, n = 19 females) or in Veh-treated group (WT, n = 21/gender; KO, n = 22 males, n = 21 females). (**C**,**D**) Cataleptic effect of Hal in male (**C**) and female (**D**) WT and KO mice (0.1 mg/kg: n = 8/genotype and gender; 0.25 mg/kg: n = 8/genotype and gender; 0.5 mg/kg: WT, n = 13 males, n = 8 females; KO, n = 13 males, n = 8 females) or Veh-treated group (WT, n = 8 males, n = 5 females; KO, n = 8 males, n = 5 females). *p < 0.05, **p < 0.01, compared with Hal-treated WT mice. (**E**) Effect of adenosine A2AR blockade by CSC treatment (2.5 mg/kg) on haloperidol-dependent catalepsy in female WT and KO mice. Animals were treated with CSC plus Hal (WT, n = 7; KO, n = 6) or Hal (WT, n = 7; KO, n = 6) and analyzed 60 min after haloperidol injection. **p < 0.01, compared with Hal-treated group. (**F,G**) Locomotor activity in male (**F**) and female (**G**) WT and KO mice induced by caffeine 7.5 mg/kg (WT, n = 11 males, n = 18 females; KO, n = 12 males, n = 18 females), compared to Veh-treated mice (WT, n = 10 males, n = 11 females; KO n = 10/gender). All data are expressed as mean ± SEM. Genotypes are as indicated.

Here we investigated the gender effect of *Rhes* deletion on behavioral responses induced by DA and adenosine drugs. At all doses tested, the DA D1R agonist, SKF 81297, similarly enhanced horizontal motor activity in males of both genotypes (Fig. 6A; three-way ANOVA with repeated measures, treatment effect: 1.25 mg/kg, F_(1,335)_ = 33.633, *p* < 0.0001; 2.5 mg/kg, F_(1,415)_ = 86.567, *p* < 0.0001). Likewise, SKF 81297 treatment in females also induced hyperlocomotion in both genotypes at either dose tested (Fig. 6B; 1.25 mg/kg, F_(1,330)_ = 10.644, *p* = 0.0018; 2.5 mg/kg, F_(1,390)_ = 14.095, *p* = 0.0003). However, female mutants showed a significant higher hyperactivity at 2.5 mg/kg drug dose (genotype effect, F_(1,390)_ = 5.456, *p* = 0.0221), compared to control treated animals.

Next, we analyzed the effect of the DA D2R antagonist haloperidol, on catalepsy. We found no major treatment effect in males following 0.1 mg/kg drug administration in both genotypes (Fig. 6C). On the other hand, both 0.25 and 0.5 mg/kg haloperidol administration induced a higher cataleptic response in male KO mice, compared to WT (Fig. 6C; two-way ANOVA, genotype effect, 0.25 mg/kg, males, F_(1,28)_ = 5.145, *p* = 0.0397; 0.5 mg/kg, males, F_(1,48)_ = 16.561, *p* = 0.0004). In contrast, in female mutants we found higher haloperidol-induced cataleptic responses at all doses tested, including the lowest one (Fig. 6D; two-way ANOVA, 0.1 mg/kg, F_(1,28)_ = 4.685, *p* = 0.0482; 0.25 mg/kg, females: F_(1,28)_ = 43.967, *p* < 0.0001; 0.5 mg/kg, F_(1,28)_ = 21.012, *p* = 0.0004).

Since activation of striatal adenosine A2AR positively modulates haloperidol-induced catalepsy^32^, we examined the effect of A2AR blockade on the exaggerated cataleptic response seen in female KO mice. Strikingly, pre-administration of the A2AR antagonist, 8-(3-Chlorostyryl)-caffeine (CSC), was able to normalize catalepsy in mutant females (Fig. 6E; two-way ANOVA, genotype × treatment interaction: F_(1,22)_ = 4.305, *p* = 0.0499).

Finally, considering that caffeine motor stimulation mainly relies on A2AR antagonism^33^,^34^, we analyzed its psychoactive properties in *Rhes* mutants. In line with enhanced A2AR response, administration of caffeine produced an overall significant higher hyperactivity in mutants of both genders (Fig. 6F,G, three-way ANOVA with repeated measures, males, F_(1,473)_ = 10.494, *p* = 0.0023; females, F_(1,583)_ = 7.140, *p* = 0.01), compared to their respective WT treated group.

### Striatal medial-to-lateral gradient of *Rhes* mRNA resembles those of *A2AR* and *D2R*

**Figure 7:**
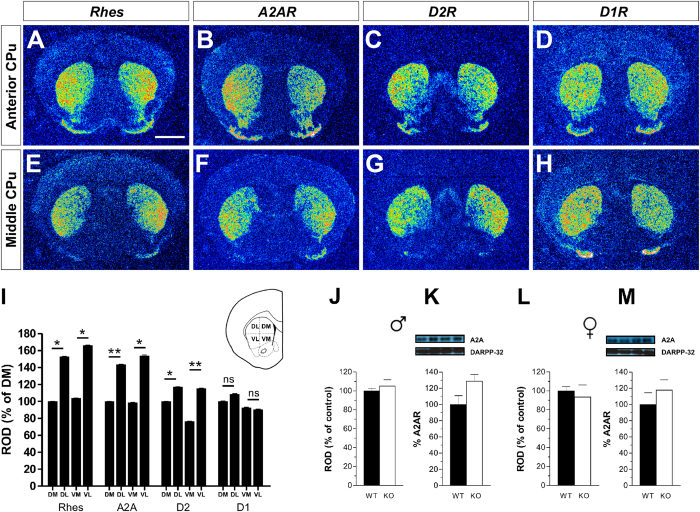
Medial-to-lateral expression gradient of *Rhes, A2AR* and *D2R* in the mouse CPu. (**A–H**) Representative pseudocolor autoradiographs from coronal section of mouse brain showing medial-to-lateral expression gradient of (**A,E**) *Rhes* mRNA, (**B,F**) *A2AR* and (**C,G**) *D2R*. (**D,H**) *D1R* mRNA appears uniformly distributed. (**I**) Histogram showing quantification of *Rhes, A2AR, D2R* and *D1R* mRNA expression levels obtained after densitometric quantification of autoradiograms. Differences are expressed as percentage of variation compared with the signal mesured in the dorsomedial part of the CPu. (**J–M**), A2AR levels were determined by radioactive ISH (n = 3/genotype for both genders; **J**,**L**) and in total striatal extracts by Western blot, in naïve male (n = 6/genotype; **K**) and female (n = 6/genotype; **M**) mice. The top panels show representative blots comparing the different genotypes. All data are expressed as mean ± SEM. *p < 0.05, **p < 0.01. Scale bar: 750 μm

To understand why the lack of Rhes impacts more profoundly on behavioral A2AR- and D2R-mediated responses, rather than those regulated by D1R, we compared the expression profile of *Rhes* mRNA to that of *A2AR, D2R* and *D1R* in the striatum of WT mice. Results showed a clear medial-to-lateral gradient of *Rhes* mRNA along the antero-posterior extent of the caudate/putamen (CPu) (Fig. 7A,E), which mirrored that of *A2AR* and *D2R* (Fig. 7B,C,F,G). Densitometric quantification of autoradiograms within the four quadrants in which the striatum was subdivided (namely, Dorso-Medial, Ventro-Medial, Ventro-Lateral, Dorso-Lateral) confirmed that Relative Optical Density (ROD) was significantly higher in the Dorso- and Ventro-Lateral quadrants as compared to the medial counterparts (Fig. 7I). Conversely, *D1R* mRNA appeared uniformly distributed within the four quadrants of the CPu nucleus (Fig. 7D,H,I).

Finally, in order to exclude that enhanced magnitude of motor responses to haloperidol and caffeine in mutants were linked to altered striatal A2AR receptor occurrence, we next assessed whether Rhes inactivation could influence *A2AR* mRNA and proteins expression levels. Notably, quantification analyses from neither radioactive ISH nor Western blot experiments showed significant differences of *A2AR* mRNA or protein levels between genotypes and genders (Fig. 7J–M, Student’s *t-*test) *p* > 0.05, per each protein, .

### Lack of Rhes does not affect hippocampal long-term potentiation

**Figure 8:**
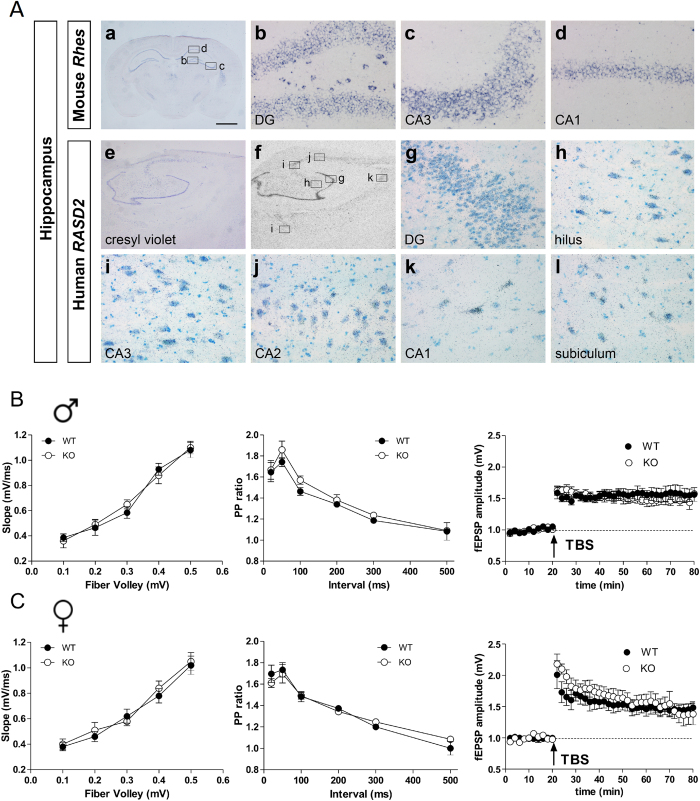
Expression pattern of *Rhes* and *RASD2* mRNA in mouse and human hippocampus. (**A**)(a–d), *In situ* hybridization experiment on a coronal section of mouse brain showing *Rhes* expression in hippocampus. Boxed regions in (a) are shown at higher magnification in (b; dentate gyrus, DG, and hilus), (c; CA3) and (d; CA1). (e-l), Nissl staining (e) and radioactive (^35^S) ISH analysis (f–l) showing expression of *RASD2* in the hippocampus in a representative section from post-mortem human brain. *RASD2* autoradiographic signal is present in the dentate gyrus (g; DG), hilus (h), CA3 (i), CA2 (j), CA1 (k), and subiculum (l). Scale bar: 1.3 mm (a); 60 μm (b–d, g–l); 1.8 mm (e,f). (**B**,**C**) Input/output relationship (left panel) and paired pulse (PP) ratios (center panel) obtained from CA1 area in WT and KO male (**B**) and female (**C**) mice. Each group represents n = 6 mice. Superimposed pooled data (right panel) representing normalized changes in the field potential amplitude (fEPSP) (± SEM) induced by TBS between male WT (n = 6) and KO (n = 4) mice (B, right panel) and female WT (n = 5) and KO (n = 5) mice (C, right panel).

In addition to striatum, *Rhes* mRNA expression is also seen in both murine and human hippocampus (Fig. 8A), but its role in hippocampal LTP is unclear. *Rhes* was expressed in the granule cell layer of the dentate gyrus (DG) and in the pyramidal cell layer of Ammon’s horn (Fig. 8A,b–d). Autoradiographic images using radioactive ^35^S ISH also showed expression of *RASD2* in the hippocampal formation of human brain samples (Fig. 8A,f). At high magnification, we observed autoradiographic grains on the granule cells of the DG and in the hilus (Fig. 8A,g,h). Pyramidal neurons of fields CA3, CA2, and CA1 of Ammon’s horn and the subiculum were also specifically labeled (Fig. 8A,i–l).

To determine whether alterations of striatal LTP in KO mice were area-specific or a widespread phenomenon associated to lack of Rhes, we measured basal synaptic transmission, short-term plasticity and LTP at CA1 hippocampal synapses in *Rhes* mutants (Fig. 8B,C). Overall, differences in the input-output relationship (*p* > 0.05 for all stimulation intensities) and in the paired-pulse ratio (*p* > 0.05 at all inter-pulse intervals) were not observed either in male (Fig. 8B) or female (Fig. 8B) mutants. In addition, theta-burst stimulation (TBS) (Fig. 8B,C), delivered to Schaffer collateral fibers, produced comparable levels of CA1-LTP in male (fEPSP amplitude at 60 min post-TBS: WT 155 ± 10%; KO 146% ± 7%, left panel) and female KO mice when compared to gender-matched controls (fEPSP amplitude at 60 min post-TBS: WT 146% ± 9%; KO 143% ± 12%; right panel). Indeed, two-way ANOVA confirmed no significant effect of gender (F_(1,16)_ = 0.146, *p* > 0.05) or genotype (F_(1,16)_ = 0.125, *p* > 0.05). Thus Rhes predominantly affects synaptic plasticity associated with striatum.

## Discussion

In the present work, we studied how lack of Rhes influences the striatal cAMP/PKA signaling-related synaptic and behavioral responses under both basal and challenged conditions, in a gender-dependent manner. We analyzed a *Rhes* KO mouse line^25^, in which the PGK-neo cassette had been removed in order to avoid any potential interference on gene transcriptional regulation^35^,^36^,^37^. In accordance with previous data^2^, we report that lack of Rhes does not affect basal motor abilities in both male and female mutant mice (data not shown). Similarly, electrophysiological data revealed that *Rhes* mutant mice display no difference in intrinsic and synaptic basal properties of striatal MSNs, when compared to controls. Moreover we did not find any apparent alteration in striatal LTD between genotypes in both genders.

Conversely, lack of Rhes was found to be critical in the expression of striatal MSNs LTP in a gender-sensitive manner. Consistently, while WT and KO males display similar LTP, in females only in a subset of D1R-positive MSNs we recorded comparable LTP amplitudes between genotypes. Based on the knowledge that LTP is a form of corticostriatal plasticity that requires intact cAMP/PKA signaling^31^, the inability of certain MSNs to potentiate EPSP amplitude in response to HFS apparently contradicts the greater striatal cAMP-PKA signaling found in *Rhes* mutant females. To explain such discrepancy, we hypothesized that the apparent loss of LTP in mutants might be generated by an exaggerated increase in PKA activity, which in turn leads to constitutive saturated LTP. Consistent with this idea, in mutant females, LFS protocol of corticostriatal fibers during baseline transmission causes significant “depotentiation” or mild potentiation in a ratio that reflects the proportion of MSNs responding to HFS with a lack of LTP or with a LTP expression similar to control, respectively.

Several striatal responses in animals and humans are affected by gender through the action of various factors, including circulating estrogens^38^,^39^,^40^. Therefore, we evaluated the potential involvement of this these sexual hormones in the expression of altered synaptic plasticity seen in female mutants. Overall, electrophysiological experiments excluded a direct implication of estrogens in determining the LTP defects as we confirmed in OVX mutants the existence of two independent striatal subpopulations of MSNs that either express LTP or undergo a saturated state. In addition, we also ruled out an altered estrogen receptor expression level as the cause for defective LTP, as striatal levels of ERα and ERβ are comparable between genotypes.

In order to identify the receptor specificity of the MSNs fraction in which LTP is saturated in KO females, we performed staining experiments after patch-clamp recordings. Remarkably, in *Rhes* mutants we found that striatal MSNs that did not express LTP are selectively A2AR/D2R-positive. Thus, in line with the notion that non-physiological phasic A2AR/cAMP/PKA responses found in female mutants may occlude activity-dependent LTP, we revealed that PKA inhibitor strikingly restores LTP in A2AR/D2R-positive KO MSNs. Moreover, we found that A2AR agonist application unmasks in WT striatum the existence of two different subsets of MSNs, one not affected by such manipulation that responded to HFS with a regular LTP, and another population of neurons, sensitive to A2AR stimulation, that fails to express a physiological LTP magnitude.

Altogether, this set of pharmacological experiments on slices from WT female striata reproduces the findings obtained in *Rhes* mutants and, in turn, demonstrates that A2AR activity drives the direction of corticostriatal plasticity in striato-pallidal pathway by regulating the magnitude of cAMP/PKA activity. In accordance to the consistent role of A2ARs in determining a physiological LTP in striato-pallidal neurons^41^, pharmacological blockade of these receptors with ZM241385 prevents HFS-induced LTP, triggering an unexpected LTD induction in WT slices. An explanation for this switch in synaptic plasticity likely relies on the ability of A2AR inhibition to unmask a higher D2R- mediated signaling which, in turn, favors LTD expression^42^. In contrast to what observed in WT, we report that ZM241385 administration in KO females prevents any form of synaptic plasticity in A2AR/D2R-expressing MSNs. This suggest that lack of LTD response in Rhes KO striata can be explained by previous results of our group demonstrating that D2R activity, as measured by GTP gamma assay, is slightly compromised in Rhes KO^2^.

It is well established that A2AR transmission exerts a pivotal role in modulating the magnitude of haloperidol-induced catalepsy and caffeine-triggered hyperactivity^32^,^33^,^34^. Therefore, in line with an exacerbated A2AR-mediated signaling in Rhes KO, we found significant higher haloperidol-induced catalepsy and caffeine-induced motor simulation responses, when compared to gender-matched controls. Although more pronounced in females, enhanced behavioral responses to haloperidol and caffeine also occurs in Rhes KO males when compared to their sex-matched treated controls. These *in vivo* observations suggest that, differently to striatal LTP and DA D1R-dependent motor stimulation, lack of Rhes affects striato-pallidal-dependent behaviors with a less prominent gender-sensitive influence. Although we reported that *Rhes* mRNA medial-lateral expression gradient strictly resembles that of *A2AR* and *D2R*, we exclude that abnormal striato-pallidal responses seen in mutants are associated to different A2AR expression since striatal mRNA and protein levels were comparable between genotypes. We also show that *Rhes* mRNA is expressed not only in striatal *Darpp*32-positive MSNs of rodents and humans, but also in various hippocampal regions. In keeping with this evidence, we demonstrated that hippocampal LTP induction in the CA1 region is unaffected in both genders of mutants, thus strengthening the sex-specific role of Rhes in modulating synaptic plasticity selectively in striatal MSNs. Furthermore, cognitive functions analyzed by object recognition and fear conditioning tasks are comparable between KO and WT mice of both genders (data not shown).

Extending our previous work^2^, the present study indicates that lack of Rhes differently impacts on striatal cAMP/PKA signaling in a gender specific fashion, causing a significant basal increase in males while generating a higher response in females, selectively under challenge conditions. In light of a gender-sensitive regulation of cAMP/PKA activity and consistent with a crucial involvement of this signaling pathway in modulating D1R-, D2R- and A2AR-mediated behavioral responses, we explain the sex-related differences in response to SKF81297, haloperidol and caffeine found in KO mice, when compared to their matched-controls.

Similarly, we argue that the striking gender-related difference in LTP expression found in A2AR/D2R-positive MSNs of mutants depends on the different magnitude of cAMP/PKA signaling triggered by HFS protocol in male and females. Therefore, we propose that yet unknown factors, other than estrogens, should be directly or indirectly involved in the regulation of striatal cAMP/PKA activity underlying the gender specific LTP expression found in Rhes KO.

Altogether our findings, indicating that Rhes plays a crucial gender-specific role in orchestrating striatal-dependent behaviors, increase the functional complexity of this GTP binding protein function.

In addition, considering the enhanced haloperidol-induced catalepsy and caffeine-triggered motor hyperactivity in mutants, we propose that *Rhes* could emerge as a novel thyroid hormone-target gene to investigate the molecular and cellular determinants associated to the use of these substances in humans.

## Methods

### Animals

In order to prevent the possibility that the presence of the PGK-neo cassette may interfere with normal transcriptional regulation within the *Rhes* locus^35^,^36^,^37^,^43^, we crossed *Rhes*^*+/loxP-neo*^ offspring^25^ with a CMV-Cre deleter strain in a B6D2 genetic background^44^. The validation of Cre-mediated loxP-flanked neo cassette excision was assessed by PCR analysis using the following primers: Rhes-EGFP_forward: 5′-CATGGTCCTGCTGGAGTTCGTGA-3′, and Rhes-Ex2_reverse: 5′-ACCACCATGCGGTAGGAGTTCT-3′. The amplification product was ~400 bp. Mice obtained were then genotyped by PCR using the following primers: Rhes-forward 5′-TTTAGGAATTTCACCTGTGT-3′ and Rhes-Ex2_reverse 5′-ACCACCATGCGGTAGGAGTTCT-3′, for *Rhes*^+/+^(WT) allele (420 bp); Rhes-EGFP_forward 5′-CATGGTCCTGCTGGAGTTCGTGA-3′ and Rhes Ex2_reverse: 5′ACCACCATGCGGTAGGAGTTCT-3′ for *Rhes*^−/−^ (KO) allele (370 bp). KO mice were viable, fertile and did not show alteration in body weight and basal motor activity (data not shown). Ten- to twelve-week-old male and female WT and KO mice, derived from mating of heterozygous animals (*Rhes*^+/−^), back-crossed to F11 generation to C57BLJ/6 strain, were used in this study. Mice were housed in groups in standard cages (29 × 17.5 × 12.5 cm) at constant temperature (22 °C ± 1 °C) and maintained on a 12/12 h light/dark cycle, with food and water *ad libitum*. Experiments were performed in conformity with protocols approved by the veterinary department of the Italian Ministry of Health and in accordance to the ethical and safety rules and guidelines for the use of animals in biomedical research provided by the relevant Italian laws and European Union’s directives (n. 86/609/EC). All efforts were made to minimize the animal’s suffering.

### Ovariectomy

In females of 2 months of age, after anaesthesia, the lumbar dorsum was shaved, and the exposed skin prepared for septic surgery (a 10% povidene–iodine scrub followed by a sterile saline wipe). The procedure was performed according to a previously published protocol^45^ with minor modifications^46^. For details see Supplementary Materials.

### Human samples

Human tissue was obtained from the Brain and Tissue Bank for Developmental Disorders at the University of Maryland, Baltimore. Samples for cryosectioning were dissected in the region of the putamen nucleus and hippocampus of brains from one male (16 years old, 16 h post-mortem) and one female (35 years old, 6 h postmortem) who had no history of primary neurological or psychiatric disorders and processed as previously described^47^.

### *In situ* hybridization (ISH)

ISH analysis was performed according to protocols previously described^48^. Digoxigenin (DIG) (Rhes, 0.5 Kb; RASD2, 2.8 Kb), fluorescein- (Darpp32, 0.6 Kb), or ^35^S-labelled (PPP1R1B, 1.5 Kb; RASD2, 2.8 Kb; Rhes, 0.5 Kb; A2AR, 1.2 Kb; Drd2, 1.0 Kb Drd1, 0.3 Kb) antisense riboprobes were used. Detailed sequences used for each probe are the following: RASD2, nucleotides 178–2971 (NM_014310.3); Darpp32, nucleotides 144–685 (NM_144828.1); PPP1R1B, nucleotides 23–1500 (NM_181505.3); A2AR, nucleotides 521–1736 (NM_009630.3); D1R^49^; D2R^49^; Rhes^25^. For double ISH on mouse tissue, sections were hybridized simultaneously with DIG- and fluorescein-labelled riboprobes. A two-step chromogenic reaction using NBT/BCIP and HNPP/Fast Red Fluorescent Detection Set (Roche) was performed to visualize the DIG- and fluorescein-labelled riboprobes, respectively. Specimens were counterstained with DAPI. For radioactive ISH, hybridized sections were exposed to Biomax MR X-ray films (Kodak) for two to four days. For each probe used, sections from different genotypes and gender were processed in parallel in order to minimize the experimental variability. Double-ISH on human tissue was performed combining DIG-labelled and^35^ S-radiolabelled riboprobes. After chromogenic reaction using NBT/BCIP for the detection of the DIG-labelled riboprobe, slides were dipped in Kodak NTB emulsion for autoradiography as previously described^47^. Sections were examined using brightfield and darkfield light microscopy.

### Image analysis and quantification

Quantification analyses were performed in blind and sample identity was not revealed until correlations were completed. For densitometric analyses, 15–20 sections per animal along the whole rostro-caudal extent of the striatum were used and images of autoradiography films resulting from radioactive ISH experiments were scanned at a resolution of 1200 dpi. For the analysis of *Rhes, A2AR, D2R* and *D1R* mRNA expression in the striatum of WT animals the Caudate-Putamen (CPu) was divided into four quadrants, dorso-medial (DM), dorso-lateral (DL), ventro-medial (VM) and ventro-lateral (VL), accordingly to Harrison and LaHoste, 2006^8^. Optical Density (OD) for each gene analyzed was evaluated in the four areas of the striatum. Background OD value was determined in structures of the same section devoid of specific signal and subtracted for correction to obtain the Relative OD (ROD) value. Results were expressed as percentage increase/decrease of mRNA-expression in the DM for each probe. Data were analyzed by Student’s *t-*test. For the analysis of *A2AR* mRNA expression ROD was evaluated in whole striatum of WT and KO adult male and female mice. Results were expressed as percentage increase/decrease of *A2AR* mRNA-expression. Analysis was performed by using StatView software (version 5.0.1.0; SAS Institute).

### Electrophysiological recordings from medium spiny neurons (MSNs)

The preparation and maintenance of coronal corticostriatal slices have been previously described^50^. For details see Supplementary Materials.

#### Intracellular recordings with sharp electrodes

For the long-term potentiation (LTP) protocol, at the beginning of intracellular recordings, magnesium ions were omitted from the medium to increase the N-methyl-D-aspartate (NMDA)-mediated component of excitatory post-synaptic potential (EPSP). As conditioning high frequency stimulation (HFS) protocol to induce either long-term depression (LTD) or LTP we used three trains (3 seconds duration, 100 Hz, 20 seconds intertrain interval). During tetanic stimulation, the intensity was increased to suprathreshold levels. To induce depotentiation, low frequency stimulation (LFS) protocol (2 Hz, 10 min of total duration) was used as previously shown^29^,^51^.

#### Whole-cell patch-clamp recordings

Patch-clamp recordings were performed as previously shown^50^,^52^. Quantitative data on EPSP modifications induced by HFS are expressed as a percentage of the controls, the latter representing the mean of responses recorded during a stable period (15–20 min) before the HFS. Current–voltage relationships were obtained by applying steps of current of 50 pA in both hyperpolarizing and depolarizing direction (from −600 to 400 pA). Statistical analysis was performed using Prism 4.0 (GraphPad). Student’s *t*-test was used to compare data within group pre vs. 30 min post-HFS. Group comparisons were analyzed with two-way ANOVA. When a significant interaction was observed the data were further analyzed with Bonferroni *post-hoc* test.

### Extracellular recordings in the hippocampus

Hippocampal slices were prepared as previously described^53^. A single slice was placed on a nylon mesh, completely submerged in a small chamber (0.5 ml) and superfused with oxygenated artificial cerebrospinal fluid (ACSF) at a constant flow rate of 3 ml/min. A bipolar tungsten stimulating electrode was positioned in the CA1 *stratum radiatum* to stimulate the Schaffer collateral fibers, and extracellular field excitatory post-synaptic potentials (fEPSPs) were recorded with a glass microelectrode (2–3 MΩ) in the *stratum radiatum*. LTP was evoked by theta burst stimulation (TBS) protocol consisting of 4 trains of 5 pulses at a frequency of 100 Hz, with an inter-train interval of 200 ms. All data are presented as mean ± SEM and *n* indicates the number of slices. Statistical analysis was performed by two-way ANOVA with gender and genotype as between subject factors. Statistical significance was set at *p* < 0.05.

### Tissue processing and double immunofluorescence of striatal MSNs

Tissue processing and double immunofluorescence were performed according to protocols previously described^52^. See Supplementary Materials for detailed information.

### Behavior

*Drug-induced motor responses.* Mice were randomly assigned to 1.25, 2.5 mg/kg SKF 81297 or vehicle (Veh)-treated groups, habituated to the experimental cage (35 × 25 × 30 cm) for 60 min, and then injected with drug or Veh and placed in the same cage, as previously described^49^. The horizontal motor activity was evaluated during 10-min intervals over a 1 h-test session, through a computerized video tracking system (Videotrack, Viewpoint S.A., Champagne au Mont d’Or, France). The effect of SKF 81297 on locomotor activity (expressed in cm) was used as dependent variable and analyzed by three-way (genotype × treatment × time) ANOVA with repeated measures.

Caffeine-induced locomotor hyperactivity was performed according to a previously validated protocol^34^. Briefly, WT and KO animals were randomly assigned to caffeine (7.5 mg/kg), or Veh-treated groups. The horizontal motor activity was evaluated during 10-min intervals over a 2 h-test session. Effect of caffeine on locomotor activity (expressed in cm) was used as dependent variable and analyzed by three-way (genotype × treatment × time) ANOVA with repeated measures.

#### Catalepsy

Catalepsy in mice was assessed according to a previous protocol^2^. To evaluate the haloperidol-induced cataleptic response, 60 min before drug treatment WT and KO mice of both genders were placed one per cage for adapting to the new environment. Animals were randomly grouped to be treated, respectively, with 0.1, 0.25, 0.5 mg/kg haloperidol (Hal) or vehicle (Veh). Catalepsy was measured 30, 60 and 120 min after drug or vehicle injection, by gently placing the front limbs of the animals over a 4 cm-high horizontal bar. The intensity of catalepsy (expressed in s) was assessed by measuring the time each mouse remained with limbs completely immobile for a maximum of 300 s. Data were analyzed between genotypes, at each haloperidol dose tested, by two-way (time × genotype) ANOVA with repeated measures. Other independent groups of female mice were used to assess the effect of 8-(3-Chlorostyryl)-caffeine (CSC) in haloperidol-induced catalepsy, according to Chen *et al.*^32^. Mice were co-administered with Veh plus 0.5 mg/kg haloperidol or haloperidol plus 2.5 mg/kg CSC (administered 10 min before haloperidol injection). Catalepsy was evaluated 60 min after treatment and analyzed by two-way ANOVA, followed by Fisher’s *post-hoc* comparison.

### Western blotting

Male and female mice of both genotypes were treated with 5 mg/kg SKF 81297, 0.5 mg/kg haloperidol (Hal) or Veh, and killed by decapitation 30-min later, according to a previous protocol^49^. Normalized values of phospo-GluA1 at residue Ser845 levels were averaged and used as dependent variable. Data were analyzed between genotypes by two-way (treatment × genotype) ANOVA, followed by Fisher’s *post-hoc* comparison, when required. Striatal levels of glutamate NMDAR (GluN1, GluN2A, GluN2B), AMPAR (GluA1, GluA 2/3) subunits, as well as estrogen (α and β) and adenosine (A2AR) receptors, were analyzed in naïve WT and KO animals of both genders. Normalized values of each target were used as dependent variable and analyzed between genotypes by Student’s *t-*test. For detailed information see Supplementary Materials.

All behavioral and biochemical values are expressed as mean ± SEM. Statistics analyses were based on parametric tests as the data were normally distributed. Normality was checked with Shapiro-Wilk test (p < 0.05).

## Additional Information

**How to cite this article**: Ghiglieri, V. *et al.* Rhes influences striatal cAMP/PKA-dependent signaling and synaptic plasticity in a gender-sensitive fashion. *Sci. Rep.* **5**, 10933; doi: 10.1038/srep10933 (2015).

## Supplementary Material

Supplementary Materials

## References

[b1] Usui H. *et al.* Isolation of clones of rat striatum-specific mRNAs by directional tag PCR subtraction. J Neurosci 14, 4915–4926 (1994). 8046460 10.1523/JNEUROSCI.14-08-04915.1994 PMC6577190

[b2] Errico F. *et al.* The GTP-binding protein Rhes modulates dopamine signalling in striatal medium spiny neurons. Mol Cell Neurosci 37, 335–345 (2008). 18035555 10.1016/j.mcn.2007.10.007

[b3] Vargiu P. *et al.* The small GTP-binding protein, Rhes, regulates signal transduction from G protein-coupled receptors. Oncogene 23, 559–568 (2004). 14724584 10.1038/sj.onc.1207161

[b4] Vargiu P. *et al.* Thyroid hormone regulation of rhes, a novel Ras homolog gene expressed in the striatum. Brain Res Mol Brain Res 94, 1–8 (2001). 11597759 10.1016/s0169-328x(01)00140-1

[b5] Falk J. D. *et al.* Rhes: A striatal-specific Ras homolog related to Dexras1. J Neurosci Res 57, 782–788 (1999). 10467249

[b6] Vallortigara J., Alfos S., Micheau J., Higueret P. & Enderlin V. T3 administration in adult hypothyroid mice modulates expression of proteins involved in striatal synaptic plasticity and improves motor behavior. Neurobiol Dis 31, 378–385 (2008). 18585460 10.1016/j.nbd.2008.05.015

[b7] Vallortigara J., Chassande O., Higueret P. & Enderlin V. Thyroid hormone receptor alpha plays an essential role in the normalisation of adult-onset hypothyroidism-related hypoexpression of synaptic plasticity target genes in striatum. J Neuroendocrinol 21, 49–56 (2009). 19094093 10.1111/j.1365-2826.2008.01802.x

[b8] Harrison L. M. & LaHoste G. J. Rhes, the Ras homolog enriched in striatum, is reduced under conditions of dopamine supersensitivity. Neuroscience 137, 483–492 (2006). 16352400 10.1016/j.neuroscience.2005.08.017

[b9] Agretti P. *et al.* Ras homolog enriched in striatum inhibits the functional activity of wild type thyrotropin, follicle-stimulating hormone, luteinizing hormone receptors and activating thyrotropin receptor mutations by altering their expression in COS-7 cells. J Endocrinol Invest 30, 279–284 (2007). 17556863 10.1007/BF03346294

[b10] Harrison L. M. & He Y. Rhes and AGS1/Dexras1 affect signaling by dopamine D1 receptors through adenylyl cyclase. J Neurosci Res 89, 874–882 (2011). 21374700 10.1002/jnr.22604 PMC3077464

[b11] Subramaniam S. *et al.* Rhes, a physiologic regulator of sumoylation, enhances cross-sumoylation between the basic sumoylation enzymes E1 and Ubc9. J Biol Chem 285, 20428–20432 (2010). 20424159 10.1074/jbc.C110.127191 PMC2898300

[b12] Subramaniam S., Sixt K. M., Barrow R. & Snyder S. H. Rhes, a striatal specific protein, mediates mutant-huntingtin cytotoxicity. Science 324, 1327–1330 (2009). 19498170 10.1126/science.1172871 PMC2745286

[b13] Steffan J. S. *et al.* SUMO modification of Huntingtin and Huntington’s disease pathology. Science 304, 100–104 (2004). 15064418 10.1126/science.1092194

[b14] Baiamonte B. A., Lee F. A., Brewer S. T., Spano D. & LaHoste G. J. Attenuation of Rhes activity significantly delays the appearance of behavioral symptoms in a mouse model of Huntington’s disease. PLoS One 8, e53606 (2013). 23349722 10.1371/journal.pone.0053606 PMC3549908

[b15] Subramaniam S. & Snyder S. H. Huntington’s disease is a disorder of the corpus striatum: focus on Rhes (Ras homologue enriched in the striatum). Neuropharmacology 60, 1187–1192 (2011). 21044641 10.1016/j.neuropharm.2010.10.025

[b16] Bang S., Steenstra C. & Kim S. F. Striatum specific protein, Rhes regulates AKT pathway. Neurosci Lett 521, 142–147 (2012). 22683505 10.1016/j.neulet.2012.05.073 PMC3389258

[b17] Harrison L. M., Muller S. H. & Spano D. Effects of the Ras homolog Rhes on Akt/protein kinase B and glycogen synthase kinase 3 phosphorylation in striatum. Neuroscience 236, 21–30 (2013). 23380502 10.1016/j.neuroscience.2012.12.062 PMC3596425

[b18] Choi B. R., Bang S., Chen Y., Cheah J. H. & Kim S. F. PKA modulates iron trafficking in the striatum via small GTPase, Rhes. Neuroscience 253, 214–220 (2013). 23999124 10.1016/j.neuroscience.2013.08.043 PMC3871862

[b19] Wullschleger S., Loewith R. & Hall M. N. TOR signaling in growth and metabolism. Cell 124, 471–484 (2006). 16469695 10.1016/j.cell.2006.01.016

[b20] Santini E., Valjent E. & Fisone G. mTORC1 signaling in Parkinson’s disease and L-DOPA-induced dyskinesia: A sensitized matter. Cell Cycle 9, 2713–2718 (2010). 20581466 10.4161/cc.9.14.12323

[b21] Subramaniam S. *et al.* Rhes, a striatal-enriched small G protein, mediates mTOR signaling and L-DOPA-induced dyskinesia. Nat Neurosci 15, 191–193 (2012). 10.1038/nn.2994 PMC3267880 22179112

[b22] Ghiglieri V. & Calabresi P. Rhes, a key element of selective neuronal vulnerability in Huntington’s disease: a striatal-specific license to kill during energy metabolism failure. Mov Disord 28, 735 (2013). 23712467 10.1002/mds.25476

[b23] Picconi B. & Calabresi P. Rhes-mTORC1 interaction: a new possible therapeutic target in Parkinson’s disease and L-dopa-induced dyskinesia? Mov Disord 27, 815 (2012). 22912975 10.1002/mds.25044

[b24] Quintero G. C. & Spano D. Exploration of sex differences in Rhes effects in dopamine mediated behaviors. Neuropsychiatr Dis Treat 7, 697–706 (2011). 22128255 10.2147/NDT.S25888 PMC3225344

[b25] Spano D. *et al.* Rhes is involved in striatal function. Mol Cell Biol 24, 5788–5796 (2004). 15199135 10.1128/MCB.24.13.5788-5796.2004 PMC480889

[b26] Svenningsson P. *et al.* DARPP-32: an integrator of neurotransmission. Annu Rev Pharmacol Toxicol 44, 269–296 (2004). 14744247 10.1146/annurev.pharmtox.44.101802.121415

[b27] Roche K. W., O’Brien R. J., Mammen A. L., Bernhardt J. & Huganir R. L. Characterization of multiple phosphorylation sites on the AMPA receptor GluR1 subunit. Neuron 16, 1179–1188 (1996). 8663994 10.1016/s0896-6273(00)80144-0

[b28] Hakansson K. *et al.* Regulation of phosphorylation of the GluR1 AMPA receptor by dopamine D2 receptors. J Neurochem 96, 482–488 (2006). 16336634 10.1111/j.1471-4159.2005.03558.x

[b29] Picconi B. *et al.* Loss of bidirectional striatal synaptic plasticity in L-DOPA-induced dyskinesia. Nat Neurosci 6, 501–506 (2003). 12665799 10.1038/nn1040

[b30] Gerfen C. R. *et al.* D1 and D2 dopamine receptor-regulated gene expression of striatonigral and striatopallidal neurons. Science 250, 1429–1432 (1990). 2147780 10.1126/science.2147780

[b31] Calabresi P. *et al.* Dopamine and cAMP-regulated phosphoprotein 32 kDa controls both striatal long-term depression and long-term potentiation, opposing forms of synaptic plasticity. J Neurosci 20, 8443–8451 (2000). 11069952 10.1523/JNEUROSCI.20-22-08443.2000 PMC6773171

[b32] Chen J. F. *et al.* The role of the D(2) dopamine receptor (D(2)R) in A(2A) adenosine receptor (A(2A)R)-mediated behavioral and cellular responses as revealed by A(2A) and D(2) receptor knockout mice. Proc Natl Acad Sci USA 98, 1970–1975 (2001). 11172060 10.1073/pnas.98.4.1970 PMC29366

[b33] El Yacoubi M., Ledent C., Parmentier M., Costentin J. & Vaugeois J. M. Adenosine A2A receptor knockout mice are partially protected against drug-induced catalepsy. Neuroreport 12, 983–986 (2001). 11303773 10.1097/00001756-200104170-00024

[b34] Lindskog M. *et al.* Involvement of DARPP-32 phosphorylation in the stimulant action of caffeine. Nature 418, 774–778 (2002). 12181566 10.1038/nature00817

[b35] Dhar V., Nandi A., Schildkraut C. L. & Skoultchi A. I. Erythroid-specific nuclease-hypersensitive sites flanking the human beta-globin domain. Mol Cell Biol 10, 4324–4333 (1990). 2370867 10.1128/mcb.10.8.4324-4333.1990 PMC360980

[b36] Pasqualetti M. *et al.* A Hoxa2 knockin allele that expresses EGFP upon conditional Cre-mediated recombination. Genesis 32, 109–111 (2002). 11857792

[b37] Pham C. T., MacIvor D. M., Hug B. A., Heusel J. W. & Ley T. J. Long-range disruption of gene expression by a selectable marker cassette. Proc Natl Acad Sci USA 93, 13090–13095 (1996). 8917549 10.1073/pnas.93.23.13090 PMC24051

[b38] Becker J. B. Gender differences in dopaminergic function in striatum and nucleus accumbens. Pharmacol Biochem Behav 64, 803–812 (1999). 10593204 10.1016/s0091-3057(99)00168-9

[b39] Castner S. A. & Becker J. B. Sex differences in the effect of amphetamine on immediate early gene expression in the rat dorsal striatum. Brain Res 712, 245–257 (1996). 8814899 10.1016/0006-8993(95)01429-2

[b40] Riccardi P. *et al.* Sex differences in the relationship of regional dopamine release to affect and cognitive function in striatal and extrastriatal regions using positron emission tomography and [(1)(8)F]fallypride. Synapse 65, 99–102 (2011). 20506565 10.1002/syn.20822 PMC2965297

[b41] Shen W., Flajolet M., Greengard P. & Surmeier D. J. Dichotomous dopaminergic control of striatal synaptic plasticity. Science 321, 848–851 (2008). 18687967 10.1126/science.1160575 PMC2833421

[b42] Calabresi P. *et al.* Abnormal synaptic plasticity in the striatum of mice lacking dopamine D2 receptors. J Neurosci 17, 4536–4544 (1997). 9169514 10.1523/JNEUROSCI.17-12-04536.1997 PMC6573334

[b43] Tuan D., Solomon W., Li Q. & London I. M. The “beta-like-globin” gene domain in human erythroid cells. Proc Natl Acad Sci USA 82, 6384–6388 (1985). 3879975 10.1073/pnas.82.19.6384 PMC390720

[b44] Johansson I., Martensson G., Nystrom U., Nasic S. & Andersson R. Lower incidence of CMV infection and acute rejections with valganciclovir prophylaxis in lung transplant recipients. BMC Infect Dis 13, 582 (2013). 24325216 10.1186/1471-2334-13-582 PMC3878887

[b45] Eddy C. A. Experimental surgery of the genital system. In: Methods of animal experimentation, Gay WI, Heavner JEH, eds. Research surgery and care of the research animal. Orlando, FL: Academic Press 7, 191 (1986).

[b46] Riazi K. *et al.* Sex and estrus cycle differences in the modulatory effects of morphine on seizure susceptibility in mice. Epilepsia 45, 1035–1042 (2004). 15329066 10.1111/j.0013-9580.2004.69903.x

[b47] Pasqualetti M. *et al.* Distribution and cellular localization of the serotonin type 2C receptor messenger RNA in human brain. Neuroscience 92, 601–611 (1999). 10408609 10.1016/s0306-4522(99)00011-1

[b48] Migliarini S., Pacini G., Pelosi B., Lunardi G. & Pasqualetti M. Lack of brain serotonin affects postnatal development and serotonergic neuronal circuitry formation. Mol Psychiatry 18, 1106–1118 (2013). 23007167 10.1038/mp.2012.128

[b49] Napolitano F. *et al.* Role of aberrant striatal dopamine D1 receptor/cAMP/protein kinase A/DARPP32 signaling in the paradoxical calming effect of amphetamine. J Neurosci 30, 11043–11056 (2010). 20720111 10.1523/JNEUROSCI.1682-10.2010 PMC6633484

[b50] Bagetta V. *et al.* Rebalance of striatal NMDA/AMPA receptor ratio underlies the reduced emergence of dyskinesia during D2-like dopamine agonist treatment in experimental Parkinson’s disease. J Neurosci 32, 17921–17931 (2012). 23223310 10.1523/JNEUROSCI.2664-12.2012 PMC6621675

[b51] Errico F. *et al.* Higher free D-aspartate and N-methyl-D-aspartate levels prevent striatal depotentiation and anticipate L-DOPA-induced dyskinesia. Exp Neurol 232, 240–250 (2011). 21946266 10.1016/j.expneurol.2011.09.013

[b52] Bagetta V. *et al.* Dopamine-dependent long-term depression is expressed in striatal spiny neurons of both direct and indirect pathways: implications for Parkinson’s disease. J Neurosci 31, 12513–12522 (2011). 21880913 10.1523/JNEUROSCI.2236-11.2011 PMC6703266

[b53] Nistico R. *et al.* Inflammation subverts hippocampal synaptic plasticity in experimental multiple sclerosis. PLoS One 8, e54666 (2013). 23355887 10.1371/journal.pone.0054666 PMC3552964

