# Supplementary material for: Rhes influences striatal cAMP/PKA-dependent signaling and synaptic plasticity in a gender-sensitive fashion
Source: Sci Rep. 2015 Jul 20;5:10933. doi: 10.1038/srep10933 (PMC4507147; doi:10.1038/srep10933)
Supplement: Supplementary Materials [file srep10933-s1.doc]

**Rhes influences striatal cAMP/PKA-dependent signaling and synaptic plasticity in a gender-sensitive fashion**

**Abbreviated title:** Rhes modulates striatal plasticity

Veronica Ghiglieri^1,2,*^, Francesco Napolitano^3,4,*^, Barbara Pelosi^5,*^, Chiara Schepisi^2,6^, Sara Migliarini^5^, Anna Di Maio^3^, Valentina Pendolino^2^, Maria Mancini^2^, Giuseppe Sciamanna^2,7^, Daniela Vitucci^3,8^, Giacomo Maddaloni^5^, Carmela Giampà^2^, Francesco Errico^3,4^, Robert Nisticò^2,6^, Massimo Pasqualetti^5,9^, Barbara Picconi^2,§,#^ & Alessandro Usiello^3,10,§,#^

^1^Department of Philosophy, Human, Social, and Educational Sciences, University of Perugia, Perugia, Italy;

^2^Fondazione Santa Lucia IRCCS, Rome, Italy;

^3^CEINGE Biotecnologie Avanzate, Naples, Italy;

^4^Department of Molecular Medicine and Medical Biotechnology, University of Naples “Federico II”, Naples, Italy;

^5^Department of Biology, University of Pisa, Pisa, Italy;

^6^Department of Physiology and Pharmacology, Sapienza University of Rome, Italy;

^7^Department of Systems Medicine, University of Rome “Tor Vergata”, Via Oxford 1, 00133 Rome, Italy;

^8^Dipartimento di Scienze Motorie e del Benessere DiSMeB, Università degli Studi di Napoli Parthenope, Naples, Italy;

^9^Istituto Italiano di Tecnologia, Center for Neuroscience and Cognitive Systems, Rovereto, Italy;

^10^Department of Environmental Sciences, Second University of Naples, Naples, Italy;

^*^ V.G., F.N. and B.Pe. share First Authorship.

# B.P. and A.U. share Senior Authorship.

**Supplementary methods**

**Ovariectomy.** In brief, skin was opened with a 1 to 2 cm incision in the midline on the lumbar vertebral line. About 1 cm to each flank, parovarian fatty tissue was identified and pulled out through a small incision. The exposed ovaries and associated oviducts were removed. Then, the skin and peritoneal tissue incisions were sutured. In sham-operated animals, the parovarian fatty tissue and ovaries were just retracted and replaced.

**Electrophysiology.**

**Intracellular recordings in the dorsolateral striatum.** Corticostriatal coronal slices were cut from male and female mice (4-6 weeks old) brains (thickness, 240 µm) using a vibratome. A single slice was transferred to a recording chamber and submerged in a continuously flowing Krebs’ solution (RT; 2.5–3 ml/min), bubbled with a 95% O_2_–5% CO_2_ gas mixture. The composition of the solution was (in mM): 126 NaCl, 2.5 KCl, 1.2 MgCl_2_, 1.2 NaH_2_PO_4_, 2.4 CaCl_2_, 10 glucose, and 25 NaHCO_3_. Drugs were bath applied by switching the solution to one containing known concentrations of drugs. Total replacement of the medium in the chamber occurred within 1 minute.

*Intracellular recordings with sharp electrodes*. Electrodes were filled with 2M KCl (30–60 MΩ). Signals were recorded with the use of an Axoclamp 2B amplifier (Molecular Devices), displayed on a separate oscilloscope, stored and analyzed on a digital system (pClamp 9, Molecular Devices). Glutamatergic excitatory postsynaptic potentials (EPSPs) were evoked every 10 seconds by means of a bipolar electrode connected to a stimulation unit (Grass Telefactor).

*Whole-cell patch-clamp recordings*. Whole-cell patch-clamp recordings were performed from MSNs visualized using infrared differential interference contrast microscopy in the dorsal striatum (Eclipse FN1, Nikon) ^1,2^. For experiments performed in current clamp mode, the following solution was adopted (in mM): 120 K-gluconate, 0.1 CaCl_2_, 2 MgCl_2_, 0.1 EGTA, 10 N-(2-hydroxyethyl)-piperazine-N-s-ethanesulfonic acid (HEPES), 0.3 Na- guanosine triphosphate (GTP), and 2 Mg-adenosine triphosphate (Mg-ATP), adjusted to pH 7.3 with KOH. Recordings were made with a Multiclamp 700B amplifier (Molecular Devices), using borosilicate glass pipettes (outer diameter, 1.5 mm; inner diameter, 0.86 mm) pulled on a P-1000 Puller (Sutter Instruments). Pipette resistances ranged from 3.5 to 5 MΩ. Membrane currents were continuously monitored and access resistance measured in voltage clamp was in the range of 5–30 MΩ before electronic compensation (60–80% routinely used). Signals were amplified with a MultiClamp 700B amplifier (Molecular Devices), recorded and stored on PC using pClamp 10 (Molecular Devices). Whole-cell access resistance was 10–30 MΩ. The stimulating electrode was located in the striatum to activate corticostriatal fibers. The recording electrodes were placed within the dorsolateral striatum. All the experiments were conducted in the continuous presence of 50 µM picrotoxin. Input resistances and injected currents were monitored throughout the experiments. Variations of these parameters > 20% lead to the rejection of the experiment.

**Tissue processing and double/triple immunofluorescence of striatal MSNs.** For the immunostaining characterization of distinct populations of MSNs, substance P (SP)-positive (direct pathway) and A2A/D2-positive (indirect pathway) ^3,4^, the neurons were filled with biocytin (0.2%) during electrophysiological recordings in acute corticostriatal slices. Slices were then post-fixed overnight at +4°C with 4% paraformaldehyde in saline solution and then stored in 0.1 M phosphate buffer (PB) with sodium azide 0.02% at 4°C. The sections were leaved in PB-TX-100 0.3% overnight at 4°C and then incubate with streptavidin-Cy3 (cod. S6402 Sigma) diluted 1:600 in PB-TX-100 0.3% for 2 h at room temperature to verify the presence of cells filled with biocytin. The sections containing cells successfully filled with biocytin were pre-incubated with a primary antibody mouse anti-A2A receptor (A2A,1:200, Millipore cod. 05-717) in 0.1 M PB, containing Triton X-100 1% and azide 0.02%, overnight at room temperature and then transferred at 4°C for two night. Subsequently, sections were rinsed three times for 15 min in 0.1 M PB and incubated with Cy2 anti-guinea pig (Jackson Immunoresearch) for 2 h at room temperature. For triple label immunofluorescence for SP, D2R and Biocytin, the sections with the biocytin-positive cells were pre-incubated with a primary antibody cocktail of guinea pig polyclonal anti-SP (1:400, Immunological Science) and rabbit polyclonal anti-A2a (1:250, Alexis cod. SA654) or rabbit anti-D2 in 0.1 M PB, containing Triton X-100 0.5 % and azide 0.02%. Section were then rinsed three times for 15 min at room temperature in 0.1 M PB and then incubated with a cocktail of Cy5 anti-guinea pig and alexa Fluor 488 anti-rabbit; or Cy5 anti-rabbit and alexa Fluor 488 anti-guinea pig for 2 h at room temperature.

The secondary antibody was diluted at 1:200. Following three additional rinses, sections were mounted on gelatin-coated slides, coverslipped with GEL-MOUNT™ and examined under an epi-illumination fluorescence microscope (Zeiss Axioskop) and a CLSM (Zeiss LSM700) was used to acquire the images.

**Western Blotting.** The heads of mice were immediately immersed in liquid nitrogen for 5–6 s. The brains were removed and the striata dissected out within 20 s on an ice-cold surface, sonicated in 1% SDS and boiled for 10 min, as previously described ^5^. This extraction procedure prevents protein phosphorylation and dephosphorylation, hence ensuring that the level of phosphoproteins measured *ex vivo* reflects the *in vivo* situation ^6^. Aliquots (2 µl) of the striatal homogenate were used for the protein determination by Bio-Rad Protein Assay kit (Bio-Rad). Equal amounts of total proteins (30 µg) for each sample were loaded onto 10% polyacrylamide gels. Proteins were separated by SDS-PAGE and transferred overnight to membranes (Immobilon PVDF Membrane, Millipore). The membranes were immunoblotted using selective antibodies against: phospo-GluA1 at residue Ser845 (1:1000; PhosphoSolution), GluN1/N2A (1:1000; Sigma), GluN2B (1:500; Cell Signaling), GluA1 (1:500; Millipore), GluA2/3 (1:500; Upstate), ERα (1:1000; Thermo Scientific), ERβ (1:500; Zymed Laboratories), A2AR (1:500; Millipore). Blots were then incubated with appropriate horseradish peroxidase-conjugated secondary antibody and target proteins were visualized by ECL detection (GE Healthcare), followed by quantification through Quantity One software (Bio-Rad). To estimate the total amount of loaded proteins, were used antibodies against GAPDH (1:1000; Santa Cruz Biotechnology) for phospo-GluA1 and estrogen receptors, DARPP-32 (1:1000; Cell Signaling Technology) and tubulin (1:40000; Sigma) for adenosine receptor, and glutamate subunits, respectively.

**Drugs.** *R*(+)-SKF 81297, haloperidol and caffeine were purchased from Sigma (St. Louis, MO). 8-(3-Chlorostyryl)-caffeine (CSC) was from Tocris Bioscience. Haloperidol was dissolved in a vehicle, composed by 10% acetic acid in saline solution. The pH was brought to 6.0 with 1 N NaOH. *R*(+)-SKF 81297 and caffeine were dissolved in saline solution. CSC was dissolved in dimethyl sulfoxide (DMSO) and then diluted in saline solution to a final 2% DMSO concentration. SKF 81297, haloperidol and caffeine were administered intraperitoneally in a volume of 0.1 ml/10 g of body weight, whereas CSC, co-administered with vehicle or haloperidol, was in a volume of 0.05 ml/10 g of body weight. For electrophysiology; CGS21680 and ZM241385, obtained from Tocris (UK), were respectively dissolved in ethanol and in DMSO, and the final concentrations of both did not exceed 0.1%. Rp-cAMPs dissolved in the intracellular solution and picrotoxin dissolved in distilled water, were obtained from Sigma-Aldrich (St. Louis, MO). Drugs were bath-applied, at the desired concentrations and according to the experimental protocol, by using a perfusion system equipped with three-way taps. When applied drugs were kept throughout the experiment.

**References**

1 Bagetta, V. *et al.* Dopamine-dependent long-term depression is expressed in striatal spiny neurons of both direct and indirect pathways: implications for Parkinson's disease. *J Neurosci* **31**, 12513-12522 (2011).

2 Bagetta, V. *et al.* Rebalance of striatal NMDA/AMPA receptor ratio underlies the reduced emergence of dyskinesia during D2-like dopamine agonist treatment in experimental Parkinson's disease. *J Neurosci* **32**, 17921-17931 (2012).

3 Deng, Y. P., Lei, W. L. & Reiner, A. Differential perikaryal localization in rats of D1 and D2 dopamine receptors on striatal projection neuron types identified by retrograde labeling. *J Chem Neuroanat* **32**, 101-116 (2006).

4 Rosin, D. L., Hettinger, B. D., Lee, A. & Linden, J. Anatomy of adenosine A2A receptors in brain: morphological substrates for integration of striatal function. *Neurology* **61**, S12-18 (2003).

5 Errico, F. *et al.* Persistent increase of D-aspartate in D-aspartate oxidase mutant mice induces a precocious hippocampal age-dependent synaptic plasticity and spatial memory decay. *Neurobiol Aging* **32**, 2061-2074 (2011).

6 Svenningsson, P. *et al.* Regulation of the phosphorylation of the dopamine- and cAMP-regulated phosphoprotein of 32 kDa in vivo by dopamine D1, dopamine D2, and adenosine A2A receptors. *Proc Natl Acad Sci U S A* **97**, 1856-1860 (2000).
